# The Complete Mitochondrial Genome and Phylogenetic Analyses of To Chicken in Vietnam

**DOI:** 10.3390/genes14051088

**Published:** 2023-05-15

**Authors:** Lan Doan Pham, Thi Thanh Nhan Giang, Van Ba Nguyen, Thi Phuong Mai Pham, Thi Thu Thuy Tran, Thi Quynh Chau Nguyen, Khanh Van Nguyen, Duy Ngoc Do

**Affiliations:** 1 Key Laboratory of Animal Cell Technology, National Institute of Animal Sciences, Thuyphuong, Bac Tuliem, Hanoi 100000, Vietnam; 2 Faculty of Veterinary Medicine, Viet Nam National University of Agriculture, Hanoi 100000, Vietnam; 3 Department of Animal Science and Aquaculture, Dalhousie University, Truro, NS B2N 5E3, Canada

**Keywords:** mitochondrial genome structure, Vietnamese indigenous chicken breed, phylogeny

## Abstract

Indigenous chicken breeds have both cultural significance and economic value since they possess unique genetic characteristics that enable them to adapt to the local environment and contribute to biodiversity, food security, and sustainable agriculture in Vietnam. To (Tò in Vietnamese) chicken, a Vietnamese indigenous chicken breed, is popularly raised in Thai Binh province; however, little known is about the genetic diversity of this breed. In this study, we sequenced the complete mitochondrial genome of To chicken for a better understanding of the diversity and origin of the breed. The results of sequencing showed that the mitochondrial genome of To chicken spans a total length of 16,784 base pairs and comprises one non-coding control region (known as the displacement-loop (D-loop) region), two ribosomal RNA genes, 13 protein-coding genes, and 22 transfer RNA genes. The phylogenetic tree analyses and estimated genetic distances based on 31 complete mitochondrial genome sequences indicated that To chicken has a close genetic distance with the Laotian native chicken breed, Lv’erwu breed in China, and Nicobari black and Kadaknath breeds in India. The result of the current study might be important for conservation, breeding, and further genetic studies of To chicken.

## 1. Introduction

**Figure 1:**
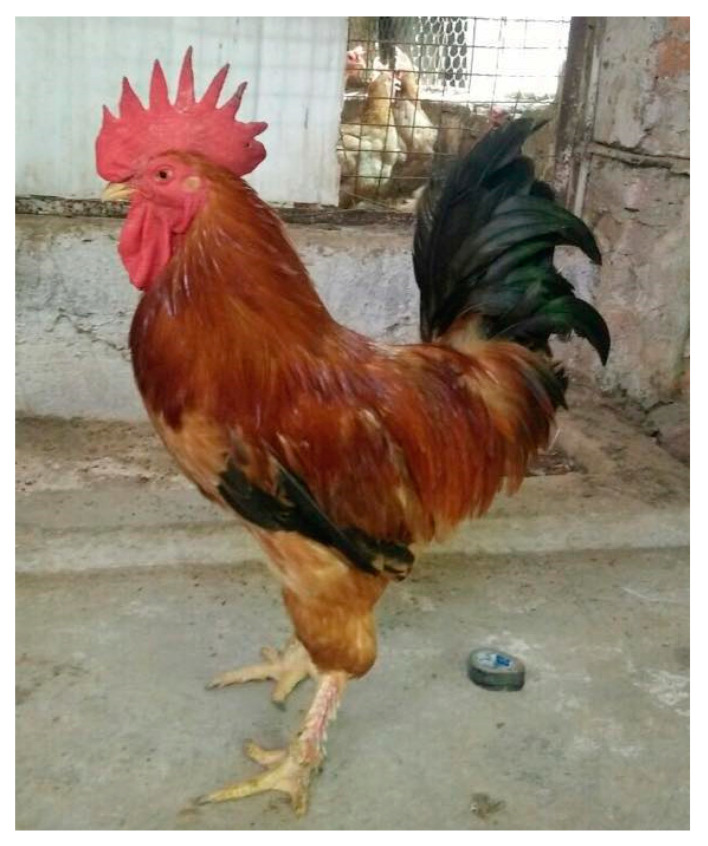
The mature To rooster.

Vietnam, located in Southeast Asia, is one of the domestication centers of wild jungle fowls from about 10,000 years ago and it is also the area of origin for several indigenous chicken breeds or ecotypes in Asia [1]. More than 30 Vietnamese native chicken breeds has been identified based on their places of origin, morphology, and conservation status [1]. Chicken is an important part of Vietnamese culture since it is commonly used in Vietnamese cuisine, and many traditional dishes feature chicken as a main ingredient. Moreover, it also plays a role in Vietnamese folklore and superstitions. For example, a rooster is considered a symbol of good luck and is believed to bring prosperity and happiness. In Vietnamese culture, it is also stated that the sound of a rooster crowing can ward off evil spirits and bring blessings to the household. Moreover, it is used as a traditional medicine to improve the health of the sick and is used for cockfighting in annual festivals. Native chickens are commonly raised in Vietnamese households for both personal consumption and for sale in local markets. For many farmers, selling chicken is an important source of income for their families. To (Tò in Vietnamese) chicken is one of the Vietnamese indigenous chicken breeds. It is originated from To village, An My commune, Quynh Phu district of Thai Binh province, belonging to Red River Dental in Northeast Vietnam. At one day old, male and female chickens have similar physical features such as milky white feathers, a golden beak, and pinkish-white legs [2]. At 19 weeks of age, the rooster has dark red feathers, a single comb, bright red wattle, yellow beak, and long yellow legs, while the hen has brown body feathers, a single comb, and a golden beak and legs [2]. To chicken has a distinct feature compared to other Vietnamese indigenous chickens as it has feathers on its legs from the feet to the junction with the thigh. In addition, To chicken leg gap has a purple-red color (Figure 1). The average age of sexual maturation is approximately 148 days, and egg yield is from 120 to 140 eggs/hen per year [3]. The body weights of roosters and hens at 19 weeks of age are about 2.2 kg and 1.8 kg, respectively [2].

To chicken has been raised for a long time and is associated with the spiritual and cultural life of the people of To village. It was used as an offer for the Kings in the past due to its high meat quality. Currently, the number of To chicken is not much and facing the risk of decreasing, so this breed has been under conservation strategies to develop valuable products such as meat and eggs, contributing to the local householder livelihoods of several communes in Thai Binh province. However, knowledge about the origin and phylogenetic relationships of To chickens with other chicken populations is still insufficient.

Recent conservation genetic studies have significant contributions to our understanding of domestication and the genetic structure of livestock through the identification of wild ancestors, answering questions relating to the extent wild ancestors contributed to the gene pool of modern livestock and how domestication events took place. Information potentially has more importance for the management and conservation of today’s animal genetic resources. Mitochondrial (mt) DNA is one of the most widely used DNA-based markers in conservation genetic studies to evaluate evolution, population genetic diversity, and the relationship of individuals. The popularity of mtDNA markers is due to several characteristics. Firstly, maternal inheritance with no recombination of mtDNA advance to trace maternal genealogies. Secondly, compared to nuclear DNA, the evolutionary rate of mtDNA is five to ten times faster, making mtDNA an ideal material for determining the difference between populations and individuals [4]. Thirdly, mitochondrial-encoded genes of mtDNA involving basic metabolic functions show that these genes are relatively conserved in adaptive processes [5]. Finally, the amplification of mtDNA is generally easier compared to nuclear DNA in PCR because of multiple copies of mtDNA per cell. 

Numerous phylogenetic studies in chickens have relied on sequences of the displacement-loop (D-loop) because this region has a higher polymorphic rate than other regions of mtDNA. Phylogenetic analyses by Liu et al. [6] classified 900 D-loop sequences of wild and domestic chickens from Eurasian into nine clades named A to I. While A, B, and E clades were widely distributed in Eurasia, others were restricted to South and Southeast Asia. These results suggest that chickens in divergent clades likely originate from different areas [6]. By analysis of the mtDNA D-loop, Oka et al. [7] revealed Japanese native chickens descended from Chinese, Korean, and Southeast Asian chickens. A study by Osman et al. [8] on the complete sequence of mtDNA D-loop region revealed the dual origin of domestic chickens in Egyptian including chickens from South Asia and the Pacific. A range of phylogenetic information of indigenous chickens of Southeast Asian countries was reported regarding mtDNA D-loop sequences. The majority of Laotian native chickens are associated with chickens from Southeast Asia and China [9]. Indonesian native chickens have three maternal lineages [10]. Most of the Thai indigenous chickens are related to chickens in South and Southwest China as well as surrounding and in Southeast Asia [11], while Philippine chickens are the origin of Pacific chickens [12]. Some phylogenetic studies of Vietnamese native chickens analyzed information of a partial sequence of the mtDNA-control region and reported that Vietnamese chickens originated from multiple maternal lineages. By using 455 base pairs (bp) sequences of mtDNA D-loop region multiple maternal origins of Vietnamese native chicken breeds including H’mong, Mia, Ri, Ho, Dong Tao, Te, Choi, Ac, Tau Vang [13] as well as Lien Minh, Dong Tao, Nhan, and Chin Cua [14] were identified. Do et al. [3] also indicated the phylogenetic relationship of To, Sau Ngon, Mong, and Tien Phong chickens with 13 haplogroups of domestic chickens in Eurasian regions based on a longer mtDNA D-loop fragment (525 bp). Ha Giang chicken population has close relationships with the silky breeds of China which is a result of the long migration across southern China [15]. However, recently, evaluations based on the complete sequence of mtDNA are predicted to be more accurate. The relatively small size of the control region limits the resolution of mtDNA. Because of the higher mutation rate of mtDNA compared to nuclear DNA, leading to high levels of repeated mutations in mtDNA which can obscure the structure of the matrilineal pedigree [16]. A study of the bird family *Icteridae* also demonstrated that the analysis of using the whole mitochondrial genome sequence better resolved and strongly supported hypotheses about phylogenetic relationships, compared to the use of phylogenetic relationships using the *Cytochrome b* and *ND2* genes [17]. Recently, several studies have achieved better analytical results when using the whole mitochondrial genome to reconstruct the domestication history of animals such as cattle [18,19,20], dogs [21], horses [22], pigs [23], and chickens [16].

In this study, the complete sequence and organization of the mitochondrial genome of To chicken were determined to support conservation, origin, and evolution studies. Furthermore, the molecular phylogenetic relationships between this chicken breed and some domestic chickens and wild fowls were revealed based on complete mtDNA sequences.

## 2. Materials and Methods

### 2.1. Sample Collection and DNA Extraction

All the sample collection was done according to the standard animal care in Vietnam. Since Vietnam does not have any specific regulations regarding animal welfare. As a result, we have adapted the Vietnamese Law on Animal Health (2015) and the Vietnamese Law on Animal Husbandry (2018) in the current research. Although both laws forbid the mistreatment of animals, they do not provide specific guidelines for animal research. To ensure the best possible practices during sample collection, the authors have additionally incorporated the guidelines for animal research based on the EU directive 2010/63. The chickens were collected from the Quynh Phu district of Thai Binh province, which was the area of origin and current distribution of To chicken. One milliliter (1 mL) of blood was collected from the wing vein and stored in vials containing Ethylenediaminetetraacetic acid (EDTA) 0.5 M as an anticoagulant at 4 °C until use. Genomic DNA was extracted from the whole blood by using GeneJET Genomic ADN Purification Kit (Thermo Scientific™, Thermo Fisher Scientific, Waltham, USA) following the manual instruction from the company and stored at −20 °C until use. The concentration of genomic DNA was measured by the Qubit^®^ 3.0 Fluorometer (Invitrogen^TM^, Thermo Fisher Scientific, Waltham, MA, USA).

### 2.2. Polymerase Chain Reaction (PCR) Amplifications of the mtDNA Sequences

A set of 23 primer pairs (Table 1) designed by Bao et al. [24] was used for the PCR reactions. The PCR reaction containing 12.5 μL buffer DreamTaq PCR Master Mix 2X (Thermo Scientific™, Thermo Fisher Scientific, Waltham, MA, USA), 0.5 μL each primer (10 pM), 100 ng DNA template, and nuclease-free water in a 25 μL reaction volume. The thermal cycling began initial denaturation at 94 °C for 3 min; followed by 35 cycles of denaturation at 94 °C for 30 s, annealing temperature (Ta) °C for 30 s (Ta °C depending on each primer pair in Table 1), extension at 72 °C for 1 min; final extension at 72 °C for 7 min. The PCR products were visualized under UV light after mixing with 6X loading dye and electrophoresis on 1.5% agarose gel stained with RedSafe^TM^ Nucleic Acid Staining Solution (iNtRON Biotechnology, Seongnam, Republic of Korea) for 35 min at 100 V.

### 2.3. Sequencing and Structure Analysis

The amplicons were sequenced in both directions using an ABI 3130 DNA analyzer (Applied Biosystems, MA, USA) with the same PCR primers. The DNA sequences were assembled using Unipro UGENE software version 40.1 [25] and exported to FASTA files. The complete mitochondrial sequence was annotated using MITOS [26]. The nucleotide composition and GC contents of To’s mitochondrial genome were calculated using DAMBE software version 7.3.5 [27]. The mitochondrial genome map of To chicken was produced by GenomeVx 2.0 [28]

### 2.4. Phylogenetic Analysis

The complete mtDNA sequence of To chicken was aligned with mitochondrial genome sequences of other chicken breeds retrieved from The National Center for Biotechnology Information (NCBI) database following accession numbers as listed in Table 2 by using the MUSCLE algorithm of MEGA software version X [29]. A maximum likelihood (ML) tree was constructed by MEGA software version X [29] using 1000 bootstrap replicates. 

## 3. Results 

### 3.1. Structure of Mitochondrial Genome of To Chicken

**Figure 2:**
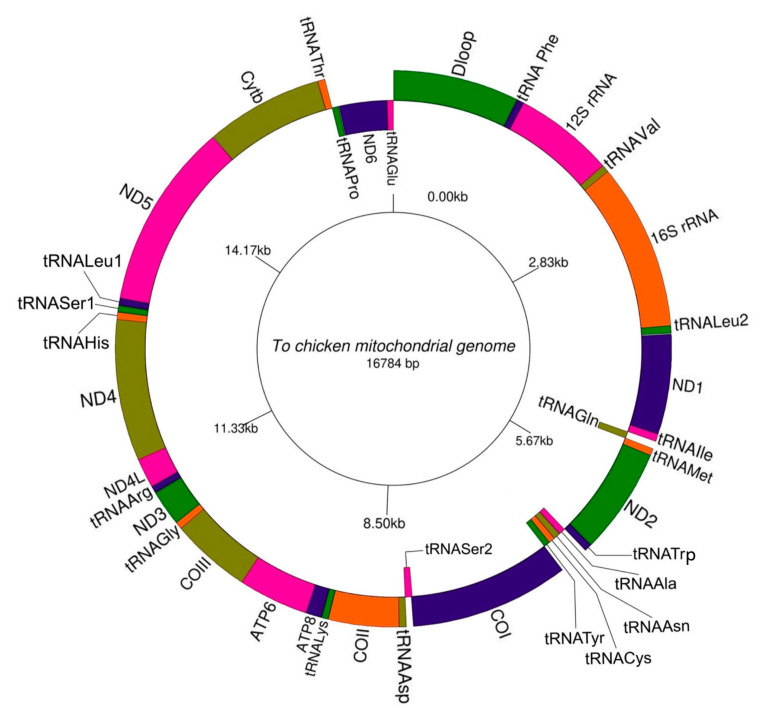
Scale drawing of the mitochondrial genome of To chicken.

The complete mitochondrial genome sequence of To chicken was determined and deposited in GeneBank with accession No. OL959342. The total length of To chicken mitochondrial genome was 16,784 bp. The nucleotide composition was 30.26% for A, 23.72% for T, 32.51% for C, and 13.51% for G. The A + T content (53.98%) is greater than G + C content (46.02%). In the genome, the GC contents have a significant impact on gene expression in chickens since the GC content in genes is responsible for approximately 10% of the variation in gene expression [30]. The structure of To mitochondrial genome was shown in Table 3. The results of annotating the structure and arrangement of the components of the mitochondrial genome of To chicken were highly similar to the typical mitochondrial genome of White Leghorn chickens identified by Desjardins and Morais [31]. The mitochondrial gene arrangements of To chicken was shown in Figure 2. Like the mitochondrial genome of other vertebrates, the mitochondrial genome of the studied chicken breed included a non-coding region (*D-loop*) and 37 genes, including 2 genes encoding ribosomal RNA (rRNA), 13 protein-coding genes, 22 genes encoding transfer RNA (tRNA), distributed on both H (Heavy) and L (Light) strands of mitochondria. The *D-loop* region and coding genes were located mainly on the H-strand except for the *nicotinamide adenine dinucleotide dehydrogenase (ND) 6* gene and eight tRNA genes on the L-strand. The start codons of the 12 protein-coding genes were mainly ATG, while that of the *cytochrome c oxidase subunits (CO) I* gene is GTG. The *COIII* and *ND4* genes ended with an incomplete stop codon (T--), under the assumption that a stop codon TAA will form after undergoing polyadenylation during post-transcription [32]; the *ND2* gene ends with a codon TAG; the *COI* gene ended with the codon AGG; and other protein-coding genes all had stop codon TAA. In the mitochondrial genome, 17 non-coding spaces were found between genes; the spaces in the length range from 1 to9 bp. While the sequences of some genes were contiguous, such as tRNA-*Ile* and *ND1*; tRNA-*Met* and *ND2*, sequences of several other genes overlapped. For example, *COI* and tRNA-*Ser^2^*, *ATP* 8 and *ATP 6*, *ND4L*, and *ND4* (Table 3). There were 7 overlaps in size 1 to 10 bp. The length of the *D loop* region was 1231 bp. The length of *12SrRNA* and *16S rRNA* were 975 bp and 1622 respectively. The overall lengths of 13 protein-coding genes were 11,394 bp and 22 tRNA genes, ranging from 65 to 76 bp in length.

### 3.2. Phylogenetic Analysis

**Figure 3:**
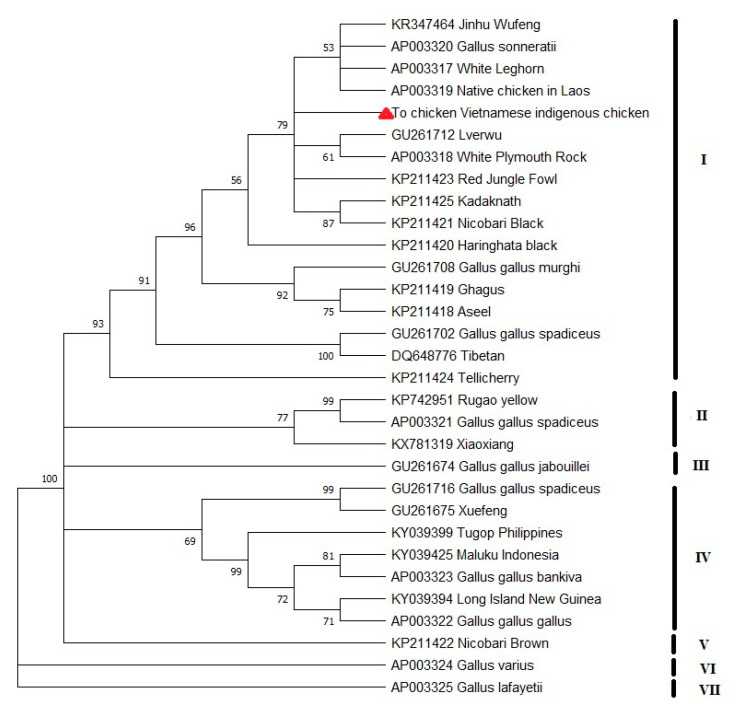
Maximum likelihood phylogenetic tree for complete mtDNA sequence of To chicken. A total of 19 sequences of domestic chicken and 11 sequences of jungle fowls. Node labels correspond to bootstrap values evaluated with 1000 replicates. Bootstrap values under 50% are not shown. The position of To chicken in the tree is marked by a red triangle.

A matrilineal phylogenetic tree was constructed based on the complete mitochondrial genome sequence of To chicken breed, 11 sequences of jungle fowls including 8 red jungle fowls sequences, 1 green jungle fowl, 1 grey jungle fowl, and 1 Ceylon jungle fowl, and 19 sequences of domestic chicken that were distributed in different areas of Asia (China, India, Laos, Philippine, Indonesia and New Guinea) and Europe taken from GenBank NCBI, which are listed in Table 2. From the results of the multi-sequence alignment, the best nucleotide substitution model was identified as HKY + G + I. The phylogenetic tree is shown in Figure 3.

The phylogenetic tree results showed seven large clades based on the complete mtDNA sequence (Figure 3). While Green Jungle Fowl (*Gallus varius*) and Ceylon Jungle Fowl (*Gallus lafayetii*) were grouped in separate clades (VI and VII respectively) and were independent with domestic chickens, and Grey Jungle Fowl (*Gallus sonneratii*) and four subspecies of Red Jungle Fowl (*Gallus gallus*): *G. g. bankiva*, *G. g. gallus*, *G. g. spadiceus* and *G. g. murghi* were grouped with domestic chickens. In the phylogenetic tree, To chicken was far from chicken groups in the island of Southeast Asia (Philippines, Indonesia, and Long Island New Guinea). To chicken was alone in a subclade and was distributed in clade I, the largest clade including most of the Indian native chicken breeds, Laotian native chickens, three Chinese native chickens, two commercial chicken breeds (White Leghorn and White Plymouth Rock), wild chicken species Grey Jungle Fowl (*Gallus sonneratii*), and Indian Red Jungle Fowl. Genetic distances analysis in this study determined To chicken had the shortest genetic distances from Grey Jungle Fowl (*Gallus sonneratii*) (0.0002386), Lv’erwu chicken (in Yunnan, China) (0.0002983), Laos native chicken (0.0004176), Nicobari black (0.0006565), and Kadaknath (0.0007162). 

## 4. Discussion

Vietnam is known for being one of the domesticated chicken centers in Southeast Asia. However, the origin and history of the domestication of Vietnamese native chickens are still uncertain. The complete mitochondrial genome sequence supplied important genetic information for accurately measuring phylogenetic relationships. In this study, the mitochondrial genome sequences and structures were determined for To chicken. The complete mtDNA genome of To chicken was 16,874 bp in size and included 13 protein-coding genes, 22 tRNA genes, two rRNA genes, and one non-coding region (D-loop region) similar to the mitochondrial genome of other vertebrates. The overall A + T contents of To chicken mtDNA genomes was 53.98% with the percentage of each nucleotide composition being 32.51% for C, 30.26% for A, 23.72% for T, and 13.51% for G which were similar to those of some Chinese native breeds such as Wuhua three-yellow chickens, Longsheng Feng chickens, Luhua chickens, Piao chickens, Taoyan chickens, and Huangshan Black chickens [33,34,35,36,37,38]. Moreover, the overall composition of Indian local chickens is in the order G > A > T > C [39]. 

The results of genetic distance analyses showed a close genetic relationship of To chicken with the native chicken breeds in Laos (Laos native chicken breed), China (Lv’erwu chicken), and India (Nicobari black and Kadaknath breeds) (Figure 3). These results were supported by the hypothesis that Vietnamese chickens have multiple maternal lineages. Previously, Vietnamese domestic chicken breeds are suggested being originated from regions with diverse maternal lineages, including Yunnan and surrounding areas in South and Southwest China, as well as neighboring countries like Burma, Thailand, and India [3,13,15]. Moreover, the phylogeny tree also demonstrated the close relationship of To chicken with the Grey Jungle Fowl (*Gallus sonneratii*), which inhabits India, north to southern Rajasthan, Gujarat, Madhya Pradesh, and Andhra Pradesh to Polavaram [40]. Meanwhile, Vietnam is a place where the domestication of Red Jungle Fowl occurred and where two subspecies *G.g.gallus* and *G.g.jabouillei* were reported inhabitants [41]. This close relationship indicated a possible contribution to the genetics of Grey Jungle Fowl into the gene pool of To chickens. Nishibori et al. [40] found molecular evidence of the inter-species hybridization between Grey Jungle Fowl and Red Jungle Fowl by phylogenetic analysis using mtDNA sequences. Meanwhile, another study also proposed evidence for the hybrid origin of domestic chickens when detected the yellow skin allele originates from Grey Jungle Fowl [42]. The Bekisar chicken, an Indonesian native chicken breed, has a hybrid origin between the Green Jungle Fowl rooster and the domesticated chicken hen [10]. This suggests the necessity to conduct further studies to test the hybrid origin of domestic chickens. 

While mtDNA serves as a useful resource to study genetic diversity and evolutionary relationships, it is essential to acknowledge its limitations in research, including incomplete lineage sorting (maternal inheritance), absence of recombination, mutational saturation, or introgression (transfer of mtDNA between species) [43,44]. Therefore, alternative genomic tools, such as whole genome sequencing, genotyping by sequencing, or utilizing different SNP panels, must be employed to analyze the genetic diversity and population structure of To chicken. Meanwhile, it is necessary to develop an efficient breeding program such as a community-based breeding program [45] for this breed to avoid the loss of its genetic diversity or enhance productivity. 

## 5. Conclusions

The complete mitochondrial genome DNA sequence of To chicken breed was determined to be 16,784 bp in length. The phylogenetic analysis suggested that To chicken has multiple maternal lineages and a close relationship with Laos native chicken breed, Lv’erwu chicken breed from China, and Nicobari black and Kadaknath chicken from India. These results will be valuable for further studies on the evolution, phylogeny, and conservation of To chicken.

## Figures and Tables

**Table 1:** Primers and conditions of amplification.

Primer	PCR Primer Sequences		Ta * °C	Expected PCR Product Length (bp)
	Forward (5′-3′)	Reverse (5′-3′)		
1	CTCGCCCTACTTGCCTTCC	TGCCTGATACCTGCTCCTTT	60	925
2	CACTGAAGATGCCAAGATGGTA	CCTTGACCTGTCTTATTAGCGA	63	748
3	TGCCAGCACAGCCTACATA	GAGACGGGTTCGCTCAAAT	58	750
4	TAGCAAGAACAACCAAGCAAAGTG	CCATTCATACAAGTCTCAATTTACGG	63	781
5	GCAAACCAAAGACCCGACTG	GTGTTCATAGATAGAAACCGACCTG	60	656
6	CGACAAGGAGGTTTACGAC	GAAGGTTTGTTAGGGTGGG	62	389
7	TTAACAGTCCTACGTGATCTGAGT	GAGTGCAATGGGAAATAATTCT	59	1077
8	TAACCACCGTCCTATTCCTG	ATCAGGCGTTGGTTATGCT	63	699
9	CGAGCGATTGAAGCCACTA	GCAAGTCGGAGGTAGAAGAAT	59	740
10	GGCTTCATGCCAAAATGACT	AGAATGGAGGAAACACCTGCTA	55.5	1109
11	CCTACTAGCCTCATCTACCGTAG	GCGTCTGGGTAATCTGAGTATC	59	1105
12	ACAGGCTTTACCCTACACCCA	GGTTAAGATGACAGTAGTGAGGATCA	60	1172
13	GCAATCCCTGGACGACTAAATCA	ATGGGCTTGGGTCAACTATGTG	62	1122
14	ACCAATAATACCATCAATCTCC	CGCTTAGTAGAAAGGATAGTGAG	59	1034
15	TTTGCCTCCTACGACTAATCAA	GCTGTATATTGTGGTGTTAGTTCATAT	54	1008
16	TCATTCGCCCTTGGACCTAT	TTGGGGTGGGTGAGTTTGAT	58	682
17	CATTCGCCCTTGGACCTATC	GATGGAAGAGTGCCTCGTTGG	63	1388
18	CCTAAAATCCCTCATTGCCTAC	TATGTTATTTGCGATGGTTAGTG	60	1157
19	GAAAGCATTGCCACCCACTGA	TGATTGCTGGGGTTCGTGTG	60	1141
20	CCACCTCCTGCCTAACCATT	TCGTCCGATGTGAAGGAAGATA	60	1020
21	AACGTACAATACGGCTGAC	AGGTTTGAGTCCTCCTTTT	61	1022
22	CCCCACAATCGGAACACTA	GGTCTAACCAAGCGGGAATA	60	735
23	GATTAGACGCCACAGCTAAA	TTCGTGAAAAGTGAGAAAGTTC	63	721

*: annealing temperature.

**Table 2:** Complete chicken mitochondrial genome deposited in the GenBank NCBI.

No.	Breed/Common Name	GenBank Accession No.	Geographic Location
1	Red jungle fowl *(G. g. bankiva)*	AP003323	Indonesia
2	Red jungle fowl *(G. g. gallus)*	AP003322	Philippine
3	Red jungle fowl *(G. g. spadiceus)*	AP003321	Laos
4	Red jungle fowl *(G. g. spadiceus)*	GU261716	Myanmar
5	Red jungle fowl *(G. g. spadiceus)*	GU261702	China
6	Red jungle fowl (*G. g. jabouillei*)	GU261674	China
7	Red jungle fowl *(G. g. murghi)*	GU261708	India
8	Red jungle fowl *(G. g. murghi)*	KP211423	India
9	Green jungle fowl *(G. varius)*	AP003324	Indonesia
10	Grey jungle fowl *(G. sonheratii)*	AP003320	Laos
11	Ceylon jungle fowl *(G. lafayettei)*	AP003325	Japan
12	White Leghorn	AP003317	Europe
13	White Plymouth Rock	AP003318	Europe
14	Lao’s negative chicken	AP00319	Laos
15	Domestic chicken	KY039399	Philippine
16	Domestic chicken	KY039425	Indonesia
17	Domestic chicken	KY039394	Long Island New Guinea
18	Aseel	KP211418	India
19	Ghagus	KP211419	India
20	Haringhata Black	KP211420	India
21	Nicobari Black	KP211421	India
22	Nicobari Brown	KP211422	India
23	Tellicherry	KP211424	India
24	Kadaknath	KP211425	India
25	Rugao Yellow	KP742951	China
26	Xiaoxiang	KX781319	China
27	Xuefeng	GU261675	China
28	Lverwu	GU261712	China
29	Jinhu Wufeng	KR347464	China
30	Tibetan	DQ648776	China

**Table 3:** Structural mtDNA of To chicken.

Gene	Strand	Position		Length (bp)	Intergenic Nucleotide *	Codons		Anti-Codon
		Start	End			Start	Stop	
*D-loop*	H	1	1231	1231	0			
tRNA-*Phe*	H	1232	1301	70	0			GAA
*12S rRNA*	H	1302	2276	975	0			
tRNA-*Val*	H	2277	2349	73	0			TAC
*16S rRNA*	H	2350	3971	1622	0			
tRNA-*Leu*^2^	H	3972	4045	74	9			TAA
*ND1*	H	4055	5029	975	0	ATG	TAA	
tRNA-*Ile*	H	5030	5101	72	5			GAT
tRNA-*Gln*	L	5107	5177	71	−1			TTG
tRNA-*Met*	H	5177	5245	69	0			CAT
*ND2*	H	5246	6286	1041	−2	ATG	TAG	
tRNA-*Trp*	H	6285	6360	76	6			TCA
tRNA-*Ala*	L	6367	6435	69	3			TGC
tRNA-*Asn*	L	6439	6511	73	1			GTT
tRNA-*Cys*	L	6513	6578	66	−1			GCA
tRNA-*Tyr*	L	6578	6648	71	1			GTA
*COI*	H	6650	8200	1551	−9	GTG	AGG	
tRNA-*Ser*^2^	L	8192	8266	75	2			TGA
tRNA-*Asp*	H	8269	8337	69	1			GTC
*COII*	H	8339	9022	684	1	ATG	TAA	
tRNA-*Lys*	H	9024	9091	68	1			TTT
*ATP8*	H	9093	9257	165	−10	ATG	TAA	
*ATP6*	H	9248	9931	684	−1	ATG	TAA	
*COIII*	H	9931	10714	784	0	ATG	T--	
tRNA-*Gly*	H	10715	10783	69	0			TCC
*ND3*	H	10784	11135	352	1	ATG	TAA	
tRNA-*Arg*	H	11137	11204	68	0			TCG
*ND4L*	H	11205	11501	297	−7	ATG	TAA	
*ND4*	H	11495	12872	1378	0	ATG	T--	
tRNA-*His*	H	12873	12941	69	1			GTG
tRNA-*Ser*^1^	H	12943	13007	65	1			GCT
tRNA-*Leu*^1^	H	13009	13079	71	0			TAG
*ND5*	H	13080	14897	1818	4	ATG	TAA	
*Cytb*	H	14902	16044	1143	3	ATG	TAA	
tRNA-*Thr*	H	16048	16116	69	0			TGT
tRNA-*Pro*	L	16117	16186	70	6			TGG
*ND6*	L	16193	16714	522	2	ATG	TAA	
tRNA-*Glu*	L	16717	16784	68				TTC

*: Overlap (−).

## Data Availability

The complete mitochondrial genome sequence of To chicken was determined and deposited in GeneBank with accession No. OL959342.
